# Quantification and reliability of [^11^C]VC - 002 binding to muscarinic acetylcholine receptors in the human lung — a test-retest PET study in control subjects

**DOI:** 10.1186/s13550-020-00634-0

**Published:** 2020-06-03

**Authors:** Zsolt Cselényi, Aurelija Jucaite, Cecilia Kristensson, Per Stenkrona, Pär Ewing, Andrea Varrone, Peter Johnström, Magnus Schou, Ana Vazquez-Romero, Mohammad Mahdi Moein, Martin Bolin, Jonathan Siikanen, Pär Grybäck, Bengt Larsson, Christer Halldin, Ken Grime, Ulf G. Eriksson, Lars Farde

**Affiliations:** 1 PET Science Centre, Precision Medicine, R&D, AstraZeneca, Stockholm, Sweden; 2 Department of Clinical Neuroscience, Centre for Psychiatry Research, Karolinska Institutet and Stockholm County Council, Stockholm, Sweden; 3 Department of Medical Radiation Physics and Nuclear Medicine, Karolinska University Hospital, Stockholm, Sweden; 4 BioPharmaceuticals R&D, AstraZeneca, Göteborg, Sweden

**Keywords:** Muscarinic acetylcholine receptors, Positron emission tomography, [^11^C]VC-002, Test-retest, Lungs

## Abstract

**Background:**

The radioligand [^11^C]VC-002 was introduced in a small initial study long ago for imaging of muscarinic acetylcholine receptors (mAChRs) in human lungs using positron emission tomography (PET). The objectives of the present study in control subjects were to advance the methodology for quantification of [^11^C]VC-002 binding in lung and to examine the reliability using a test-retest paradigm. This work constituted a self-standing preparatory step in a larger clinical trial aiming at estimating mAChR occupancy in the human lungs following inhalation of mAChR antagonists.

**Methods:**

PET measurements using [^11^C]VC-002 and the GE Discovery 710 PET/CT system were performed in seven control subjects at two separate occasions, 2–19 days apart. One subject discontinued the study after the first measurement.

Radioligand binding to mAChRs in lung was quantified using an image-derived arterial input function. The total distribution volume (*V*_T_) values were obtained on a regional and voxel-by-voxel basis. Kinetic one-tissue and two-tissue compartment models (1TCM, 2TCM), analysis based on linearization of the compartment models (multilinear Logan) and image analysis by data-driven estimation of parametric images based on compartmental theory (DEPICT) were applied.

The test-retest repeatability of *V*_T_ estimates was evaluated by absolute variability (VAR) and intraclass correlation coefficients (ICCs).

**Results:**

The 1TCM was the statistically preferred model for description of [^11^C]VC-002 binding in the lungs. Low VAR (< 10%) across analysis methods indicated good reliability of the PET measurements. The *V*_T_ estimates were stable after 60 min.

**Conclusions:**

The kinetic behaviour and good repeatability of [^11^C]VC-002 as well as the novel lung image analysis methodology support its application in applied studies on drug-induced mAChR receptor occupancy and the pathophysiology of pulmonary disorders.

**Trial registration:**

ClinicalTrials.gov identifier: NCT03097380, registered: 31 March 2017.

## Background

Molecular imaging by positron emission tomography (PET) has since long been widely applied in neuroscience to examine brain exposure and the binding of drug candidates to different targets [1]. Recently, there has been increasing interest in using molecular imaging to support drug development also for non-CNS disorders, such as pulmonary diseases. PET-examination of lung tissue has long been dominated by tumor imaging using the radiotracer 2-Deoxy-2-[^18^F]fluoro-D-glucose ([^18^F]FDG) which serves as a critical tool for the diagnosis of lung cancer and monitoring of different therapies [2, 3]. Besides the wide use of [^18^F]FDG, a series of new radioligands aimed to examine lung cancer biology and to improve tumor management has been recently introduced into clinical routine [4]. In addition, a small number of studies have been published on deposition of radiolabeled drugs in the lungs [5–7], Importantly, some attempts have also been made to measure receptor binding in the lungs, for instance beta-adrenoreceptors [8], muscarinic acetylcholine receptors (mAChRs) [9], opioid receptors [10] and epidermal growth factor receptor mutational status [11]. Taken together, those initial studies have demonstrated a promising potential of molecular imaging to support novel drug developments for pulmonary disorders.

Inhaled drug delivery is commonly used for treatment of respiratory diseases since it assures efficacy in the lung while avoiding systemic side effects. However, the determination of drug concentration in the bronchial tree has been a major challenge. Thus, there is a need to develop methods that confirm target engagement and ultimately guide the selection of appropriate dosing for inhaled drugs.

Muscarinic receptor antagonists together with other bronchodilators are currently the mainstay of pharmacotherapy for relieving dyspnoea and improving quality of life in patients with chronic obstructive pulmonary disorder (COPD) and asthma [12, 13]. Chemical modifications of the atropine molecule, a reference drug which is administered systemically, have led to the development of inhalatory compounds with longer duration of action and less side effects. This group of drugs is often referred to as long-acting mAChR antagonists (LAMA) [14]. To further optimize balance between long term efficacy and higher tolerability it is of central interest to understand the magnitude and duration of local drug exposure and the occupancy at muscarinic receptors after inhalation. Molecular imaging using PET and suitable radioligands for muscarinic receptors could serve such purposes.

Initial attempts to develop radioligands for imaging of mAChRs lead to the development of the non-selective mAChR antagonist [^11^C]VC-002 (N-[^11^C]-methyl-piperidin-4-yl-2-cyclohexyl-2- hydroxy-2-phenylacetate) by the PET-centre in Groningen, the Netherlands [15]. The pulmonary uptake of [^11^C]VC-002 has previously been examined in four healthy subjects and in one single subject also after intravenous administration of the muscarinic antagonist glycopyrronium bromide. In this subject, the pulmonary uptake was reduced by 68% suggesting that a large fraction of the pulmonary signal of [^11^C]VC-002 represents specific binding to muscarinic receptors in the lung [9].

Despite these promising initial results, the development of [^11^C]VC-002 was not then further pursued and its potential for detailed quantitative analysis examined. This temporary halt might be attributable at least in part to lack of specific interest from industry or academic research institutions at that time. However, information on the feasibility of performing full quantification in lungs is a prerequisite for the use of this methodology in further clinical research and in drug development. In a translational approach to the extended validation of [^11^C]VC-002, we recently performed a pilot PET study in non-human primates with the aim to examine and compare lung mAChR occupancy after inhalation and iv infusion of the mAChR antagonist ipratropium [16]. The results provided preliminary support that the radioligand [^11^C]VC-002 has favourable characteristics for quantitative analysis of muscarinic receptor occupancy in lungs and thus paved the way to moving to the human validation phase.

The aim of the present PET investigation in human subjects was to examine the quantification and repeatability of [^11^C]VC-002 binding to mAChR in lungs using a test-retest paradigm in order to establish the methodological platform for subsequent use in applied studies. In detail, seven healthy male subjects were recruited and examined twice on two separate days with the full experimental protocol repeated including radioligand synthesis, i.v. injection and PET/CT measurement. A prerequisite for the quantitative analysis was the advancement of methodology for automated definition of organs/regions of interest and an image derived arterial input function. In detail, the performance of region of interest based analyses, non-linear compartment modelling and the multilinear regression variant of Logan’s linear graphical analysis, which utilises a linearization of the two-tissue compartment model, was evaluated and compared for cross-validation purposes. Moreover, a previous voxel-based method for image analysis by Data-driven Estimation of Parametric Images based on Compartmental Theory (DEPICT) [17] was also evaluated after adjusting it to imaging of non-brain regions. The present methodological study constitutes the first, self-standing, preparatory study panel in a clinical trial overall aiming at estimation of muscarinic receptor occupancy in the human lungs after inhalation of mAChRs antagonists (NCT03097380). Results from the remaining study panels will be presented in a separate publication.

## Subjects and methods

The study was approved by the Regional Ethics Committee in Stockholm and the Radiation Protection Committee at the Karolinska University Hospital, Stockholm. Written informed consent was obtained from each subject.

### Subjects and design

Study participants (inclusion criteria: men, 20–50 years of age) were recruited at the PET Centre, Department of Clinical Neuroscience, Karolinska Institutet, Stockholm, Sweden. Subjects were healthy according to medical history, clinical examination, and routine laboratory blood and urine tests. No medications were used at the time of the study. PET examinations were carried out at the Department of Nuclear Medicine, Karolinska University Hospital, Solna. Design of the study period included two imaging sessions 1–4 weeks apart. In each session a low-dose CT of the chest was followed by a PET measurement using the radioligand [^11^C]VC-002. The two sessions were performed at the same time of the day. To capture any possible adverse event the study was completed by a follow-up telephone call within 1 week after the last session.

### Radiochemistry and PET/CT imaging procedures

[^11^C]VC-002 was prepared as previously described [15]. The radiochemical purity of [^11^C]VC-002 exceeded 99% at time of injection. The mean radioactivity per single injection was 220 MBq (SD ± 20 MBq, range 188–239 MBq, *N* = 7) and the molar activity was 370 GBq/micromole (SD ± 209 GBq/micromole, range 124–737 GBq/micromole). The calculated mean mass of the radioligand injected per single measurement was 0.29 μg (SD ± 0.19, range 0.1–0.62 μg, *N* = 7). At such “tracer dose” conditions there is no significant effect of chemical mass on the radioligand binding parameters.

As part of preparatory activities before imaging an arterial cannula was inserted in the radial artery of one arm and a venous cannula was inserted in each arm. The arterial cannula was used for arterial blood sampling during PET measurement. One venous cannula was used for bolus injection of radioligand (duration of injection was 10 s), whereas the other venous cannula was used for venous blood sampling for comparisons between venous and arterial data (data not shown).

The imaging measurements were performed using a GE Discovery PET/CT 710 system. The subjects were positioned in supine position. Initially, a low-dose CT scan (7.5 mAs, 120 kVp) of the chest was performed for attenuation and scatter correction purposes. This was followed by I.V. bolus injection of the radioligand [^11^C]VC-002 dissolved in a sterile physiological phosphate buffer (pH 7.4). The cannula was immediately flushed with 10 ml. saline. Emission data were acquired in 3D list mode with no respiratory gating information and were reconstructed into a consecutive time-series of 3D PET images with the following sequence of time frames: 9 × 10 s, 2 × 15 s, 3 × 20 s, 4 × 30 s, 4 × 60 s, 4 × 180 s, and 12 × 360 s, i.e. 38 frames with a total duration of 93 min. The reconstructed 4D PET image was corrected for subject movement in a retrospective, image-data-driven process whereby inter-frame subject motion was estimated in the reconstructed images. The procedure is described in detail in the supplemental material.

### Arterial blood sampling

To obtain an arterial input function, an automated blood sampling system (Allog, Sweden) was used to collect blood samples continuously during the first 10 min of each PET measurement. Furthermore, arterial blood samples (2 ml) were drawn manually at approximately 2.5, 5, 7.5, 10, 15, 20, 30, 45, and 90 min after [^11^C]VC-002 injection. Note that manual samples were taken already during automated blood sampling to have early information also on plasma radioactivity and radiometabolites. Radioactivity in 1 ml of the manually drawn samples was then immediately measured for 10 s in a well counter cross-calibrated with the PET system. After centrifugation, 0.2 ml plasma was pipetted, and plasma radioactivity was measured in a well counter. Blood samples for the measurement of radiometabolites were drawn at approximately 5, 10, 20, 30, 45, and 60 min in five of the PET-measurements and up to 30–45 min in the remaining eight PET-measurements.

### Plasma radiometabolite analysis of [^11^C]VC-002

The fraction of plasma radioactivity corresponding to unchanged radioligand in plasma was determined as has been previously described for other PET radioligands [18]. Briefly, the plasma samples were deproteinized with acetonitrile and analyzed by high-performance liquid chromatography (HPLC) with radiodetection. In detail, an ACE C-18 HPLC column (ACE, 5 μm, 50 × 250 mm) was eluted at 5 ml/min with a mixture of acetonitrile (A) and aqueous ammonium formate (0.1 M) (B) according to the following gradient: 0–4.0 min (A/B) 40:60; 4.0–6.0 min (A/B) 80:20; 6.1–7.0 min (A/B) 90:10; 7.1–8.1 min (A/B) 40:60.

#### Image processing and analysis

Image data processing and analysis consisted of the following overall steps: (1) PET and CT image pre-processing, (2) automated delineation of lung and other regions of interest, (3) derivation of the arterial plasma input curve, (4) inspection of time curves in blood and lungs, (5) quantitative analysis of radioligand binding in lungs, (6) evaluation of test-retest repeatability of binding parameters.

#### Image pre-processing

For each PET measurement, the series of consecutive images were integrated to obtain a summation PET image showing average radioactivity concentration during the entire measurement (0–93 min). To control for within subject positioning differences between baseline (test) and follow-up (retest) measurements, the summation PET images were used to obtain co-registration parameters. Images of the follow-up measurements were subsequently resliced using these co-registration parameters (see below). In preparation for combined PET-CT image processing the CT images were resliced from the original 0.98 × 0.98 × 3.27 mm voxel size to the 3.65 × 3.65 × 3.27 mm voxel size of the PET images.

#### Delineation of regions of interest (ROI)

For each PET/CT measurement, ROIs corresponding to the lungs were delineated through an automated procedure using both the PET and the resliced CT images. A detailed description of the delineation procedure is included in the supplemental material.

#### Derivation of arterial plasma input curve

The ROI of the aortic arch was applied to extract the time-(radio)activity curve (TAC) representing radioactivity in whole blood. The curve was interpolated to 1 s resolution. In addition, the whole blood and plasma radioactivity concentration, measured in the collected manual arterial samples, was used to calculate the ratio of plasma to whole blood radioactivity. Preliminary evaluation indicated that this ratio was essentially constant throughout the PET measurement. By consequence, the average ratio across all time points with manual blood sampling was used to multiply the continuous, interpolated image-derived whole blood TAC with the average plasma-over-blood ratio to obtain the TAC of total radioactivity in plasma to be used as input function for quantification. Worth noting is that radiometabolite correction was not performed, i.e. the total plasma TAC was used in the quantification of radioligand binding based on the assumption that radioactively labelled metabolites also enter the lung tissue [16].

#### Description of the time curves for [^11^C]VC-002 in blood and lungs

The basic examination of [^11^C]VC-002 binding in lungs involved visual inspection of the distribution of radioactivity, inspection of TACs in arterial whole blood, plasma, and lungs, as well as calculation of the ratio between the TACs of lung and plasma, or, between plasma and whole blood. Besides the thereby obtained plasma-to-blood ratios, estimates based on measured haematocrit values were also obtained and compared to the ones based on direct plasma measurements. Additionally, the metabolism of [^11^C]VC-002 was assessed from the results of HPLC analysis of arterial plasma.

#### Quantitative analyses of [^11^C]VC-002 binding

The lung ROI was applied to the series of PET images to extract the corresponding TAC. The TAC was analysed and interpreted according to a compartment model describing the pharmacokinetics of radioligand in lung tissue. For details on the underlying compartment model used in lungs please refer to the supplement that also shows a schematic view of the model (see Supplemental Fig. 5). The main outcome measure was the total distribution volume (*V*_T_), which is equivalent to the ratio of the radioactivity concentration in the lung to the total plasma concentration at equilibrium. *V*_T_ has the unit of ml/cm^3^: i.e. it gives the volume of blood (in milliliter) required to match the amount of radioligand molecules present in one unit of the lung volume (in cm^3^). Multiplying the *V*_T_ value with the total volume of the target organ will give the milliliter of plasma necessary to account for the total amount of radioligand in that organ. Total binding (*V*_T_) of [^11^C]VC-002 in lung was estimated using the one- and two-tissue compartmental model (1TCM and 2TCM, respectively), and the multi-linear variant of Logan’s linear graphical approach (MLLogan) was employed [19–21]. The 1TCM provided estimates of 2 kinetic rate constants (*K*_1_, *k*_2_) and *V*_T_ was derived using the expression *V*_T_ = *K*_1_/*k*_2_. The 2TCM provided 4 kinetic rate constants (*K*_1_, *k*_2_, *k*_3_, *k*_4_) and *V*_T_ was derived using the expression *V*_T_ = *K*_1_/*k*_2_ × (1 + *k*_3_/*k*_4_).

To account for the radioactivity contained in blood vessels in lungs the whole lung TACs were fitted using a version of 2TCM that, besides the kinetic rate constants also estimated the fractional blood content (*V*_b_) as well as the time-shift between blood/plasma and tissue radioactivity curves. Subsequently, *V*_b_ and the time-shifted input curve was used to match the tissue signal. Note that the PET images and/or TACs were not corrected for fractional air content since respiratory gating information and the necessary specific CT sequences were not acquired.

Besides ROI-based quantification, binding parameters were also obtained at the voxel level. For this purpose, data-driven estimation of parametric images based on compartmental theory (DEPICT) was employed as described in the literature and using the software toolbox available from the author [17]. Traditional kinetic modelling, such as the 1TCM and 2TCM described above, operates on the basis of a priori fixed number of tissue compartments to describe the observational data. In contrast, DEPICT does not work with an a priori fixed number of kinetic tissue compartments but estimates the optimal number of compartments, referred to as model order (MO), from the data itself; hence, it is a data-driven approach. DEPICT allows for a more detailed analysis of lung data that exhibits inhomogeneity in terms of tissue composition, fractional air and blood content. The approach was confined to perform the voxel-wise estimation only in voxels within the body yet excluding those in or near the heart and liver (for details, see the “Description of the derivation of ROI masks” in the supplemental section). The range of the exponents used for the derivation of the basis function table for DEPICT were logarithmically spaced between 0.0034 1/min (i.e. 10% of the isotope half-life for ^11^C) and 0.6 1/min [17]. The DEPICT approach provided detailed parametric images of the fractional blood content (*V*_b_), the rate constant describing transfer from plasma to tissue (*K*_1_), total binding (*V*_T_), as well as the MO for each voxel. Finally, the lung ROIs were applied to the parametric images to obtain the mean (or in case of MO, the median) parameter for all voxels within the organ.

#### Evaluation of repeatability of binding estimates

Absolute variability (VAR) was calculated as the difference between the test and retest parameters (e.g. *V*_T_) values divided by the mean of these two and multiplied by 100 to get percentage values [22]. The VAR was first calculated for each subject and then summarized across subjects to obtain descriptive statistic parameters. In addition, the intra-class correlation coefficient (ICC) was calculated [23].

#### Evaluation of the time-stability of binding estimates

The time stability of [^11^C]VC-002 binding parameters, and test-retest repeatability of V_T_ was calculated for different durations of the TAC data entered into the quantification. The values for the total, 90-min data were used as a point of reference to evaluate results for shorter durations. VAR was summarized across subjects using median statistic due to the low number of subjects.

### Statistics

Descriptive statistics, quantitative analyses and computations for test-retest analysis were performed using the MATLAB, version R2014b (www.mathworks.com). The Akaike information criterion [24] and *F* test were used to identify the compartment model that provided the statistically preferred interpretation of the data. The *F* test was 1-tailed (right). Pearson’s correlation coefficient was used to analyse the association between *V*_T_ values obtained by different approaches. In all analyses, the statistical significance (alpha level) was set at *p* = 0.05.

## Results

Seven subjects, all men, mean age 37 years (range 24 to 48 years) completed this part of the clinical study. For six subjects the two PET examinations were carried out 2–19 days apart whereas one subject discontinued after the first PET examination. Quantitative analyses were performed on all seven subjects, whereas the test-retest statistical analysis was performed on the six subjects who completed the study.

### Description of the time curves for [^11^C]VC-002 in blood and lungs

The PET images were corrected for motion artefacts (c.f. Methods). The more elaborate process of correction, involving repeated PET image reconstruction, was required only in one measurement (subject #5, retest).

**Fig. 1:**
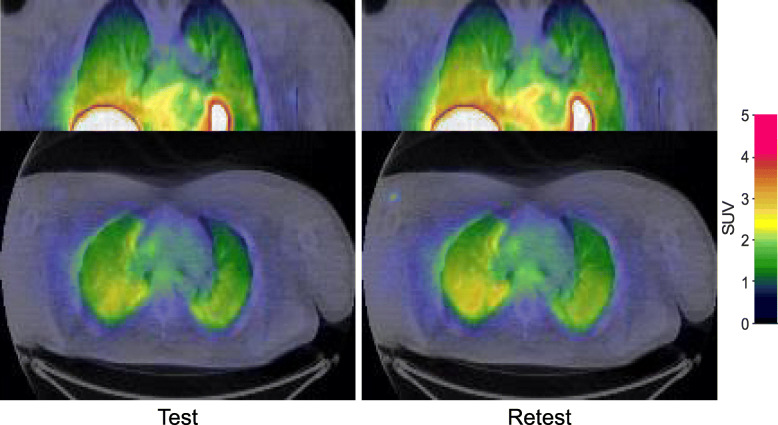
Coronal and axial summation PET images (0–93 min) in thorax after intravenous injection of the radioligand [^11^C]VC-002 in healthy subject #1. Coloured PET images in standardized uptake value units (SUV) are overlaid on structural CT images in grey scale

Following intravenous injection of [^11^C]VC-002, the radioactivity was rapidly distributed throughout the lungs (Fig. 1). Radioactivity was highest in the posterior-inferior and lowest in the anterior, apical region of the lungs as explained by the large differences in fractional air content. Note also the very high radioactivity levels in the heart and liver (noticeable at the bottom in the frontal sections), which was due to high density of mAchR’s in myocardium and hepatobiliary elimination, respectively. Furthermore, there is a visual impression that the PET and CT image are out of register. This apparent mismatch is likely due to the aforementioned gradient in pulmonary PET signal from the basal toward the apical regions, and the fact that the outer boundaries of the lungs in the PET and CT images were blurred to differing degrees in lack of respiratory gating.

**Fig. 2:**
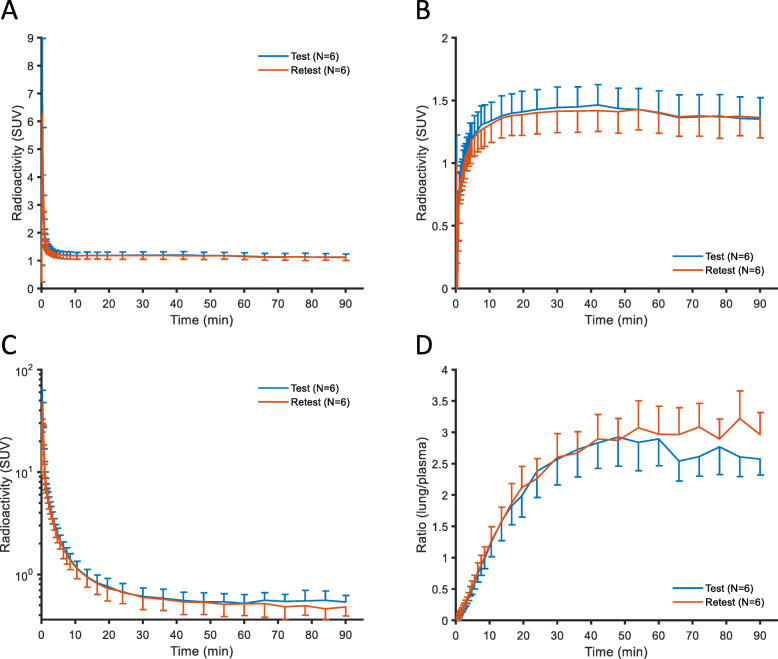
**a** Time-radioactivity curves (TACs, mean ± SD) in standardized uptake value units (SUV) for the lung after i.v. injection of [^11^C]VC-002. **b** TACs in lung, corrected for radioactivity in pulmonary blood volume. **c** TACs in plasma. **d** Lung-to-plasma ratio of TACs

The TACs for total lung radioactivity peaked within 20 secs after injection and remained roughly constant thereafter (Fig. 2a, individual curves shown in Supplemental Fig. 6). However, the blood-volume-corrected lung TACs increased over 20 min and declined slowly towards the end of the measurement (Fig. 2b, individual curves shown in Supplemental Fig. 6). There was a rapid decrease in total plasma radioactivity during the first 10 min. Thereafter, the decrease was slow (Fig. 2c, individual curves shown in Supplemental Fig. 6). The ratio of radioactivity between whole lung tissue and plasma reached a plateau level after about 50 min (Fig. 2d).

The plasma-to-blood ratio of radioactivity concentration for [^11^C]VC-002 was 1.71 ± 0.14 (mean ± SD, *N* = 7, 90 min) and at a constant level during examination time (data not shown). This value and its time stability are expected if the radioligand does not enter blood cells. Assuming that [^11^C]VC-002 indeed behaves in this manner the hematocrit was also used to estimate the plasma-to-blood ratio. The estimated ratio was 1.80 ± 0.10 (mean ± SD, *N* = 7) and the individual values correlated significantly with the ratios calculated from arterial and plasma samples (*R* = 0.61, *R*^2^ = 0.38, *p* = 0.026, data not shown).

**Fig. 3:**
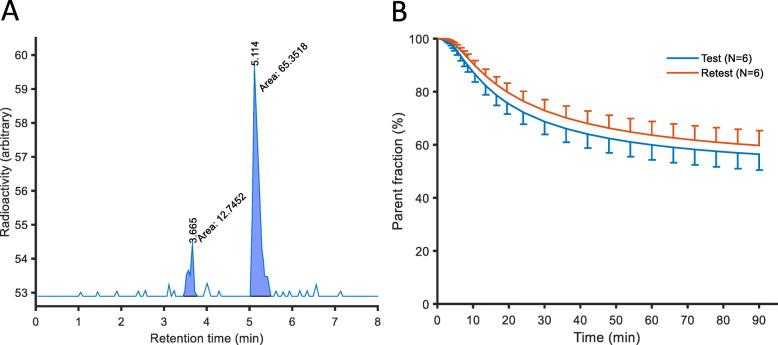
**a** Radiochromatogram from gradient HPLC analysis of human plasma at 30 min after I.V. injection of [^11^C]VC-002 in subject #1. The larger peak to the right represents unchanged [^11^C]VC-002, whereas the left peak represents a radioactively labelled metabolite that is more polar than [^11^C]VC-002. **b** Time course for the fraction (%) of radioactivity in plasma representing unchanged [^11^C]VC-002 (mean fitted curves with SD indicated using error bars). An empirical model using the Richards equation [25] was fitted to the measured parent fraction

The parent compound in plasma could be identified in the HPLC analysis (Fig. 3). In addition, there were peaks for radioactive metabolites that were more polar than the parent compound. The unchanged fraction of [^11^C]VC-002 was 69 ± 10% (mean ± SD, *N* = 6) at 30 min and 60 ± 11% (mean ± SD, *N* = 6) at 60 min after radioligand injection.

### Quantitative analyses of [^11^C]VC-002 binding

**Fig. 4:**
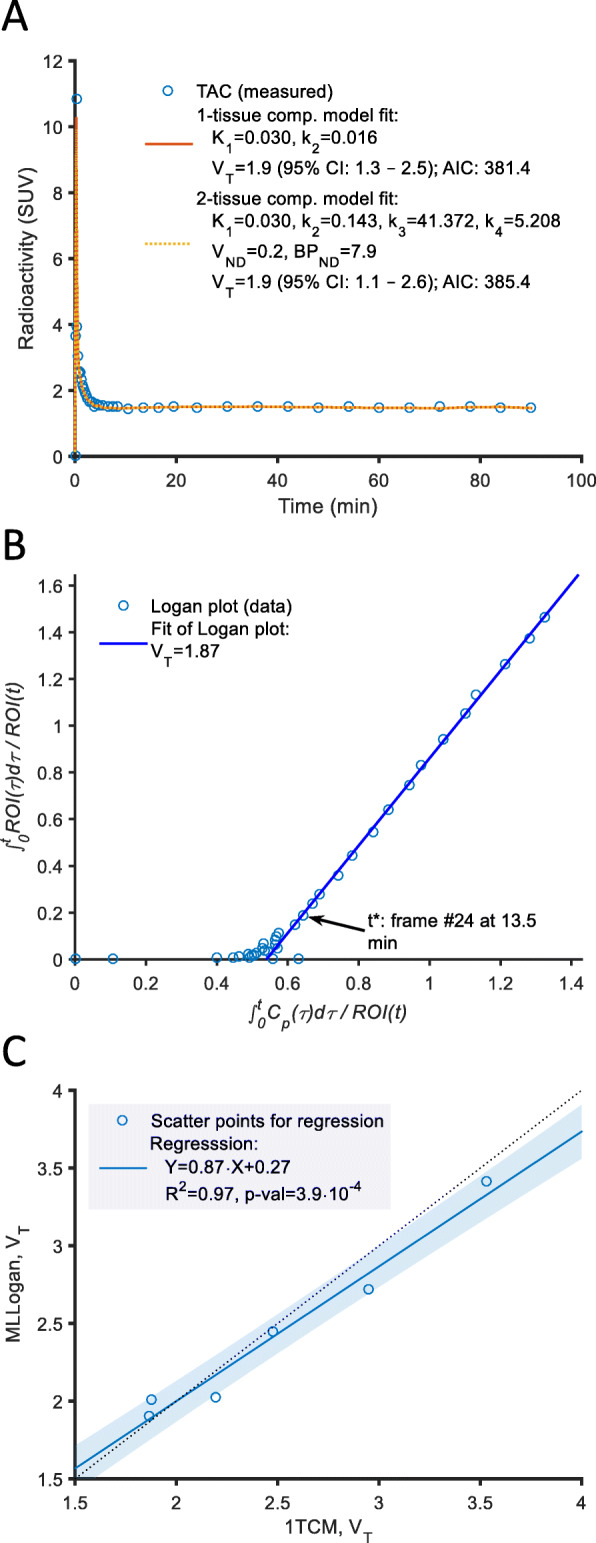
**a** Radioactivity in lung tissue vs time (not corrected for blood volume) after I.V. injection of [^11^C]VC-002 in subject #1 and 1TCM, 2TCM fits. **b** Illustration of Logan’s linear graphical analysis for subject #1 (1st PET). **c** Regression of V_T_ parameter estimates from MLLogan vs. 1TCM

The lung TACs for [^11^C]VC-002 were interpreted using compartmental and linear graphical analyses (Fig. 4a, b). The models were fitted to experimental data using the arterial plasma curve as input function. The compartment models converged to a solution in all cases in terms of providing a model curve closely fitting the experimental data (Fig. 4a). Logan’s linear graphical analysis indicated that a linear phase was reached early after injection, Fig. 4b).

The individual rate constants obtained by the one-tissue compartment model (1TCM) and the two-tissue compartment model (2TCM) for the repeated PET measurements are given in Supplemental Table 1. The rate constant *K*_1_ was similar for the two models and ranged from 0.02 to 0.05 ml/cm^3^/min. The rate constant *k*_2_ ranged from 0.01 to 0.02 1/min for the 1TCM, and from 0.03 to 0.20 1/min for 2TCM. The rate constants *k*_3_ and *k*_4_ obtained by the 2TCM varied considerably between individuals and could in most cases not be reliably estimated. According to the AIC and F statistics, the 1TCM was the preferred model for description of lung TAC data (Supplemental Table 1). In the ROI-based analysis, the value of fractional blood content (*V*_b_) in the lung varied between 17 and 27% at the test measurement (22 ± 4% mean ± SD, *N* = 6, Supplemental Table 1).

There was a statistically significant linear correlation between *V*_T_ parameters estimated by 1TCM and MLLogan (Fig. 4c). The individual *V*_T_ values obtained by the 1TCM ranged from 1.8 to 3.8 ml/cm^3^ (2.7 ± 0.7 ml/cm^3^ mean ± SD, *N* = 7) whereas *V*_T_ values obtained by MLLogan analysis ranged from 2.0 to 3.5 ml/cm^3^ (2.7 ± 0.6 ml/cm^3^ mean ± SD, *N* = 7, Supplemental Table 1).

The total injected chemical mass of the radioligand was between 0.10 and 0.62 μg. There was no systematic effect of chemical mass on *V*_T_ (data not shown).

### DEPICT analysis

**Fig. 5:**
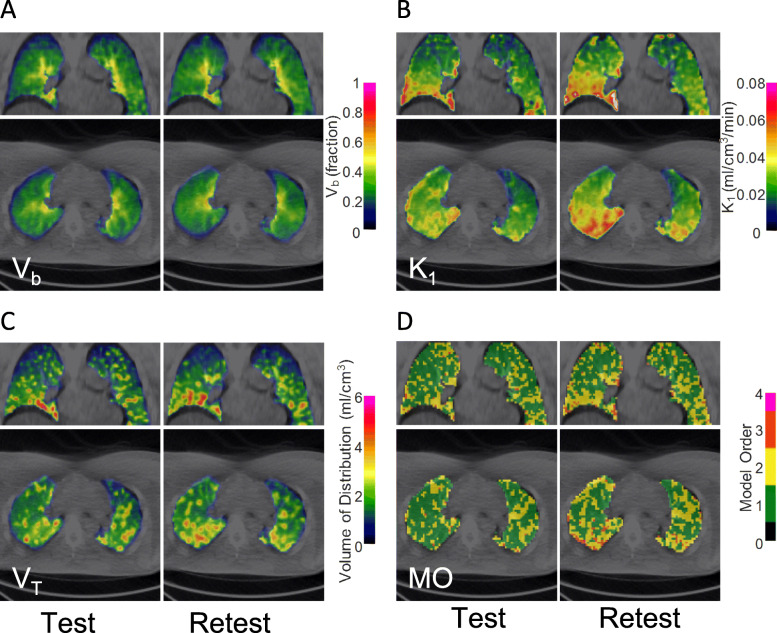
Representative parametric lung images obtained from DEPICT analysis of [^11^C]VC-002 binding in subject #1. Note that the heart and liver were not included in the generation of parametric images. **a** Fractional blood volume (*V*_b_). **b** Kinetic rate constant *K*_1_, showing the radioligand influx rate from blood to tissue that is proportional to blood flow and the radioligand extraction coefficient. **c** Total distribution volume (*V*_T_), which is the main outcome parameter of the analysis. **d** Compartmental model order (MO), showing the estimated number of pharmacokinetic compartments in tissue that is sufficient to adequately describe the data

The voxel-wise analysis generated images showing heterogeneity or gradients for the four parameters across lung (Fig. 5). As expected, the fractional blood volume, *V*_b_, followed a gradient of increasing blood volume from the lung periphery to hilar regions (Fig. 5a). The *K*_1_ and *V*_T_ parametric images displayed a vertical gradient with lowest values in the apex and highest values in the basal part of the lung (Fig. 5b, c). The estimated number of kinetic compartments, model order (MO) was 1 for most pulmonary voxels but higher typically close to the boundaries or at the base of the lungs (Fig. 5d).

In the DEPICT analysis, the value of *V*_b_ in the lung ROI varied between 5 and 24% at the test measurement (15 ± 6% mean ± SD, *N* = 6) across subjects and was in each case lower than the estimates from ROI-based analysis (Supplemental Table 1). The values for the kinetic rate constant *K*_1_ in the lung ROI were close to the ones obtained by 1TCM, with an average of 0.03 ± 0.01 ml/cm^3^/min (mean ± SD, *N* = 6), range 0.02–0.04 ml/cm^3^/min. The within-lung average *V*_T_ values were 2.4 ± 0.6 ml/cm^3^ (mean ± SD, *N* = 6), which is excellent agreement with the *V*_T_ values obtained by the ROI-based 1TCM or MLLogan analyses (Table 1 and Supplemental Table 1). The median value of the MO parameter in the lung ROI was typically 1, i.e. a single tissue compartment could best describe the TACs for most voxels.

**Table 1:** Summary statistics of test-retest PET measurement analysis using 90-min TAC^1^ duration (*N* = 6)

Model	Mean ± SD *V*_T_ (test)	Mean ± SD *V*_T_ (retest)	Mean (min–max) VAR (%)	Median VAR (%)	ICC^5^
1TCM^2^	2.5 ± 0.7	2.7 ± 0.6	11.2 (0.1–22.3)	11.3%	0.86
MLLogan^3^	2.4 ± 0.6	2.7 ± 0.6	12.1 (1.8–28.1)	10.1%	0.86
DEPICT^4^	2.4 ± 0.6	2.7 ± 0.6	12.4 (1.1–24.4)	13.3%	0.91

^1^TAC time-(radio)activity curve, VAR absolute variability, SD standard deviation

^2^1TCM one-tissue compartment model

^3^MLLogan multi-linear variant of Logan’s graphical analysis

^4^DEPICT data-driven estimation of parametric images based on compartmental theory

^5^ICC intraclass correlation coefficient

### Test-retest metrics

Detailed results on test-retest metrics are presented for analyses of the full, 90 min data acquisition (Table 1). In this sample of 6 subjects the mean *V*_T_ was a few percent higher at retest. The mean VAR was similar and varied between 11 and 12% across the methods and the ICC also indicated good repeatability (Table 1).

### Time stability of *V*_T_

**Fig. 6:**
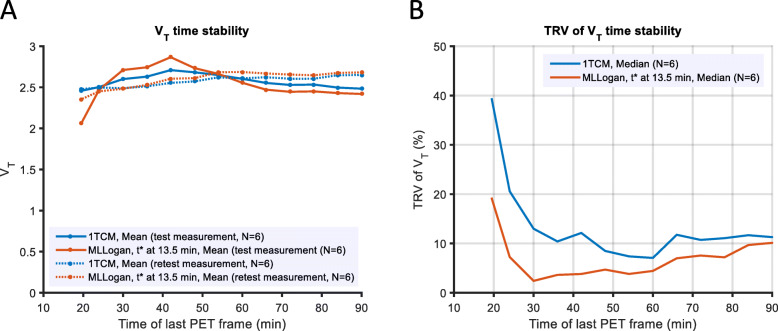
Time stability of *V*_T_ measurements for [^11^C]VC-002. The effect of progressive truncation of measurement time on: **a** the lung *V*_T_ mean estimates, and **b** their repeatability (absolute variability, VAR). Comparison of time stability between one-tissue compartment model (1TCM) and multi-linear Logan graphical analysis (MLLogan)

V_T_ estimates were overall stable from 50 to 90 min duration of the TACs (Fig. 6a). When decreasing the TACs used for analysis below 45 min, the *V*_T_ estimates by MLLogan were more impacted then those by 1TCM.

The repeatability of 1TCM and MLLogan was similar with about 10% median VAR when using the total 90 min of acquired PET data (Table 1, Fig. 6b). Progressive truncation of the TACs entered the quantitative analysis improved the repeatability for both methods with lowest overall VARs obtained when 50–60 min of PET data were used for the 1TCM and 30 min for MLLogan. Accordingly, detailed test-retest metrics were additionally calculated for the 60 min TACs (Table 2).

**Table 2:** Summary statistics of test-retest PET measurement analysis using 60-min TAC^1^ duration (*N* = 6)

Model	Mean ± SD *V*_T_ (test)	Mean ± SD *V*_T_ (retest)	Mean (min–max) VAR (%)	Median VAR (%)	ICC^4^
1TCM^2^	2.6 ± 0.8	2.6 ± 0.7	9.4 (4.8–16.8)	7.1%	0.89
MLLogan^3^	2.6 ± 0.6	2.7 ± 0.7	7.8 (1.3–24.6)	4.4%	0.90

^*1*^*TAC* time-(radio)activity curve, *VAR* absolute variability *SD* standard deviation

^*2*^*1TCM* one-tissue compartment model

^*3*^*MLLogan* multi-linear variant of Logan’s graphical analysis

^*4*^*ICC* intraclass correlation coefficient

## Discussion

The radioligand [^11^C]VC-002 has previously been examined in an initial pilot study in humans [8]. The present study provided a thorough examination of the suitability of [^11^C]VC-002 for quantitative PET-measurements of radioligand binding to muscarinic receptors in the human lung. In a test-retest paradigm, six control subjects were examined twice on separate days using [^11^C]VC-002

The test-retest repeatability of the total volume of distribution (*V*_T_) was dependent of the duration of the TACs entered into the analysis. The *V*_T_ obtained with MLLogan, at 60 min was highly repeatable with a median VAR of 4.4% and an ICC of 0.90. When entering extended TACs the *V*_T_ values were less repeatable which likely is due to inclusion of image frames with more severe subject movements towards the end of the measurement. It can be concluded that the repeatability of [^11^C]VC-002 binding is in the range of widely used PET radioligands for brain imaging [26–28]. The repeatability of [^11^C]VC-002 binding confirms that the radioligand should be suitable for quantification of changes in lung mAChR binding in relation to drug administrations.

Lung imaging data poses several challenges when compared with established quantitative approaches developed for neuroimaging since the 1980s. Favourable conditions in neuroimaging are the typically limited occurrence of motion-related issues, artefacts, the typical absence of polar radioactive metabolites in tissue, and, in some cases, access to a reference region devoid of specific binding but having a similar level of non-specific binding as the target regions. Such conditions are not present in lung imaging and a prerequisite for the present quantitative analysis was adaptation or advancement of previous methodology.

In previous studies on receptor binding in lung the analysis mostly has been based on manually drawn ROIs that do not necessarily cover the entire lung volume [7, 9]. The methodology here presented of delineating lung (and aortic) regions in a more automated fashion offers a full coverage of the lung volume according to the anatomical boundaries and enables evaluation without a user-dependent bias which would potentially be present if regions were drawn manually.

A challenge for quantitative PET imaging of the lung is posed by respiratory movements as well as voluntary movement artefacts related to the long data acquisition time. A major potential improvement for future studies would be to implement state-of-the-art methodologies using cardiac and respiratory gating information. The second challenge was partially addressed in the present study by placing pillows under the knees and arms to offer a more comfortable position on the couch. However, these steps did not fully ameliorate subject movements. Instead, the methodology used here was directed towards minimizing the effect of voluntary subject movements by a correction based on frame-by-frame realignment.

A major advantage for quantitative PET imaging of the lungs is that large vessels, as well as the heart, are in a central position within the field of view. In the present study, the arch of the aorta was selected to derive the time curves for radioactivity in arterial whole blood directly from the PET images. In other studies, the ascending aorta has been used [29]. However, primarily due to the high level of [^11^C]VC-002 binding in myocardium, the arch of the aorta is advantageous since it should not be subject for spill-in from heart tissue.

Information on the time curve for the concentration of radioactivity in arterial whole blood was available from three sources: the automated arterial blood sampling system for the first 5 min post injection, manual arterial blood samples (2.5–90 min) and, directly from the PET image, in the ROI of the aortic arch as discussed above. The image-derived curve was chosen to represent whole blood radioactivity all throughout the PET measurement primarily since it is the practical approach to be preferred for future and larger clinical studies.

The plasma-to-blood ratio for [^11^C]VC-002 exhibited stability over time suggesting that there was no or negligible entrance into or binding to blood elements. The ratios estimated from the hematocrit was close to and correlated significantly with the ratios calculated from arterial and plasma samples (Fig. 2d). Accordingly, (venous) haematocrit measurements may potentially be used to estimate the plasma-to-blood ratio instead of labour demanding centrifugation of whole blood and direct measurements of radioactivity in whole blood and plasma samples.

Radioligand metabolism was moderate (about 60% unchanged 1 hour after injection) but still higher than the low levels of metabolism reported in the early study from the PET-center in Groningen, NL [9]. A contributing factor to the discrepancy may be due to differences in the analytical methods (column, eluent, flow rate etc.) used in the two studies. Such differences may affect separation of radioligand metabolites from parent compound and insufficient resolution may lead to an over-estimation of parent radioligand. Furthermore, the absence of online detection of radioactivity eluting from the HPLC column in the previous study resulted in poor temporal resolution between radiochemical entities (1 mL fractions were collected after the HPLC column) and thus increased the risk of co-eluting radioactive metabolites with the parent. Importantly, initial evaluations indicated that a plasma input function not corrected for radioactive metabolites provided better fits than a corrected curve (data not shown). Since the lung does not have a blood-tissue barrier like that in brain, it is plausible that radioactively labelled metabolites may enter the lung parenchyma. Accordingly, the uncorrected input function was used in the quantitative analyses.

The radioligand [^11^C]VC-002 has high affinity to mAChRs (*K*_d_ < 0.1 nM) [14] and the muscarinic receptor density in the lungs is high (21–165 fmol/mg protein) [30], suggesting that specific binding to the lung mAChRs may be quantified. Several approaches are used to determine specific binding. Firstly, a reference region devoid of target receptors may be used in quantitative analysis. However, there is no reference region that would permit direct, simplified calculation of specific binding in lung tissue. Another approach to differentiate non-specific from specific binding purely from imaging data at baseline has been to use the rate constants k_3_ and k_4_ obtained with the 2TCM. This approach has been applied to established radioligands such as [^11^C]raclopride [31]. Yet, in the present study, 1TCM was statistically preferred and the 2TCM yielded highly variable, poorly identified values for *k*_3_ and *k*_4_. Thus, an alternative, experimental approach to understand the relative contribution of non-specific and specific binding would be to treat subjects with an unlabelled molecule that blocks virtually all muscarinic binding sites. Such experimental data, obtained previously, suggest that the major component of radioactivity signal (up to 70%) emanating from the lung represents specific binding [9]. Thus, specific binding represents the major component of *V*_T_, total binding parameter that was used in the present study analysis. Importantly, even if full blockade cannot be achieved in practice, pre-treatment experiments may still be useful for separating contribution of specific and non-specific binding, as well as for determining the in vivo affinity of [^11^C]VC-002 to muscarinic receptors, if the inhibition (receptor occupancy curve) can be sufficiently characterised through several experiments with possibly different doses of the pre-treatment drug [16].

The kinetic rate constants *K*_1_ and *k*_2_ obtained with the 1TCM were low, suggesting that only 2–3% per minute of the radioligand enters or exits lung tissue. These slow rates are consistent with the tissue-only TACs which display no distinct peak, but rather a slow radioligand entry into the lung tissue and minimal exit within the timeframe of image acquisition (Fig. 2b). An explanation for this slow rate of radioligand kinetics in lungs may be that [^11^C]VC-002 is a positively charged molecule, a known requirement for muscarinic agonists and antagonists [32, 33]. Development of suitable F-18-labelled radioligands is required to follow radioactivity over longer time.

Voxel-wise quantification of [^11^C]VC-002 binding was carried out using the DEPICT method. The resulting parametric images confirmed the expected inhomogeneity in lungs and in general concurred with ROI-based analysis that overall a one-tissue compartmental structure is the preferred model for characterization of binding kinetics in lungs. The DEPICT analysis tested in the present study may be useful for clinical studies, e.g. in patients with chronic obstructive pulmonary disease or interstitial lung diseases where patchy pattern of affected lung tissue brings extra challenges [34–36].

## Conclusions

The study results suggest that PET imaging of muscarinic receptor binding in lung using the radioligand [^11^C]VC-002 is feasible and that a 60-min acquisition time allows for calculation of binding parameters which appear to be robust. Furthermore, [^11^C]VC-002 provides conditions allowing for generation of an image derived input function and haematocrit values can be used to build an arterial plasma input function, which may simplify routine studies in clinical research. The test-retest repeatability of [^11^C]VC-002 appears to be sufficient for demonstration of target engagement, dose finding and comparison of drug formulations in experiments on drug induced mAChR receptor occupancy and on the pathophysiology or treatment of pulmonary disorders. In short, the kinetic behaviour and good repeatability of [^11^C]VC-002 as well as the novel lung image analysis methodology support its application in the subsequent panels of the present clinical trial or in other future studies.

## Supplementary information

**Additional file 1.** Supplemental Material 1

## Data Availability

The datasets used and/or analysed during the current study are available from the corresponding author on reasonable request.

## Abbreviations

PET: Positron emission tomography
CNS: Central nervous system
[^18^F]FDG: 2-Deoxy-2-[^18^F]fluoro-D-glucose
mAChR: Muscarinic acetylcholine receptor
COPD: Chronic obstructive pulmonary disorder
LAMA: Long-acting mAChR antagonist
[^11^C]VC-002: N-[11C]-methyl-piperidin-4-yl-2-cyclohexyl-2-hydroxy-2-phenylacetate
DEPICT: Data-driven Estimation of Parametric Images based on Compartmental Theory
CT: Computerised tomography
SD: Standard deviation
I.V.: Intravenous
HPLC: High-performance liquid chromatography
ROI: Region of interest
TAC: Time-(radio)activity curve
*V*_T_: Total distribution volume
1TCM: One-tissue compartmental model
2TCM: Two-tissue compartmental model
MLLogan: Multi-linear variant of Logan’s linear graphical approach
*V*_b_: Fractional blood content
MO: Model order
VAR: Absolute variability
ICC: Intra-class correlation coefficient
