# Supplementary material for: Quantification and reliability of [11C]VC - 002 binding to muscarinic acetylcholine receptors in the human lung — a test-retest PET study in control subjects
Source: EJNMMI Res. 2020 Jun 3;10:59. doi: 10.1186/s13550-020-00634-0 (PMC7270393; doi:10.1186/s13550-020-00634-0)
Supplement: Supplementary file 1 — Additional file 1. Supplemental Material 1 [file 13550_2020_634_MOESM1_ESM.pdf]

SUPPLEMENTAL MATERIAL

QUANTIFICATION AND RELIABILITY OF [^{11}C]VC-002 BINDING TO MUSCARINIC ACETYLCHOLINE RECEPTORS IN THE HUMAN LUNG – A TEST-RETEST PET STUDY IN CONTROL SUBJECTS

Zsolt Cselényi,[1, 2] Aurelija Jucaite,[1, 2] Cecilia Kristensson,[4] Per Stenkrona,[2] Pär Ewing,[4] Andrea Varrone,[2] Peter Johnström,[1, 2] Magnus Schou,[1, 2] Ana Vazquez-Romero,[2] Mohammad Mahdi Moein,[2] Martin Bolin[3], Jonathan Siikanen[3], Pär Grybäck[3], Bengt Larsson[4], Christer Halldin,[2] Ken Grime,[4] Ulf G Eriksson,[4] Lars Farde[1, 2]

[1]*PET Science Centre, Precision Medicine, R&D, AstraZeneca, Stockholm, Sweden;*
[2]*Department of Clinical Neuroscience, Centre for Psychiatry Research, Karolinska Institutet and Stockholm County Council, Stockholm, Sweden;*
[3]*Department of Medical Radiation Physics and Nuclear Medicine, Karolinska University Hospital, Stockholm, Sweden;*
[4]*BioPharmaceuticals R&D, AstraZeneca, Göteborg, Sweden*

Corresponding author:

Zsolt Cselényi
PET Science Centre, Precision Medicine, R&D, AstraZeneca
Email: Zsolt.Cselenyi@astrazeneca.com

SUBJECTS AND METHODS

Procedure for correction of subject movement between frames

1. The series of time frames of the attenuation-corrected reconstructed PET images was inspected visually. Movement artefacts were identified on time frames and also aided by comparison to the CT image obtained just before PET image acquisition. An example of such artefacts is shown in Supplemental Figure 1.

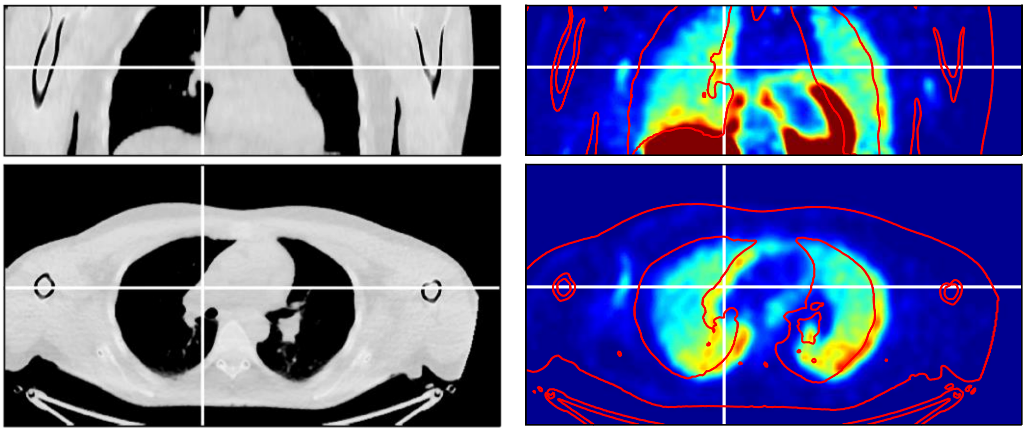

Supplemental Figure 1. Reconstruction artefacts due to subject motion. Left: frontal and horizontal CT images for subject #5, second (retest) PET measurement (radiological convention). Right: corresponding PET images for the 32nd time frame (51-57 min post injection). Red contours indicate contrast edges obtained from the CT image, and the mismatch of the lung surface boundaries between CT and PET. The mismatch results in reconstruction artefacts that are visible as bands of apparently higher radioactivity levels outside the CT defined boundaries, i.e. on the left side of the subject).

2. If no obvious artefacts were detected by visual inspection the PET images were evaluated frame-by-frame to estimate six parameters for subject realignment (translation and rotation,). In detail:

    The first time frames corresponding to 0-3 minutes post injection (p.i.) were excluded from the evaluation since they were dominated by high vascular radioactivity levels thus confounding the movement estimation. The time frames between 4-5 min p.i. were integrated and smoothed with a Gaussian filter with 10 mm full-width-at-half-maximum (FWHM). 2-2 horizontal image slices at the bottom and the top of the smoothed image, respectively, were cropped (discarded). Assuming that there was no significant movement during the first five minutes, the resulting image was then used as the reference to obtain the 6 movement realignment parameters for each of the later time frames. The 3 translation and 3 rotation parameters describing the movement between later frames and the reference image were sequentially estimated using the co-registration algorithm in SPM12 (https://www.fil.ion.ucl.ac.uk/spm/software/spm12/).

    To obtain valid movement estimates 2-2 horizontal image slices at the bottom and the top of the smoothed image, respectively, were cropped (discarded) for the following reasons. When the coregistration algorithm is searching for the right set of movement realignment parameters it iteratively reslices the source image (i.e. the one for which the realignment parameters are to be estimated) into the space of the target image with a gradually updated set of parameters. At each iteration it then calculates an information criterion, such as the mutual information measure, to determine the degree of agreement between the images. The

criterion is expected to be at its maximum when the right set of coregistration parameters are found. In the current context of coregistering thoracic PET images, however, this algorithm may break down since all major thoracic organs (lungs, heart and liver) with high radioactivity levels were partially outside the field of view (FOV). Thus, without applying cropping to the target image these organs in the source images could likely be resliced into the space of the target image in such a way that spatial locations just *outside* the FOV in the source image (and thus with *no* available radioactivity information!) would end up *inside* the FOV of the target image when searching for the right set of parameters. The information criterion would then underestimate the degree of agreement and prevent the algorithm from finding the right set of parameters. Cropping the target image ensured that the likelihood of this artefact was reduced. According to quality checks of the results of the current dataset there were no such artefacts.
An example of subject movement estimates is shown in Supplemental Figure 2.

A.

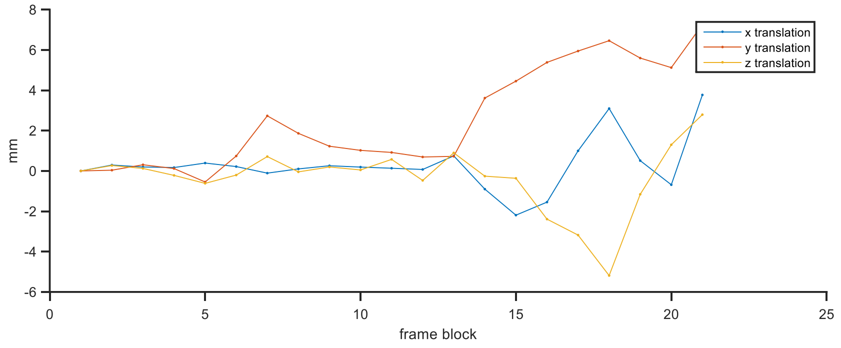

B.

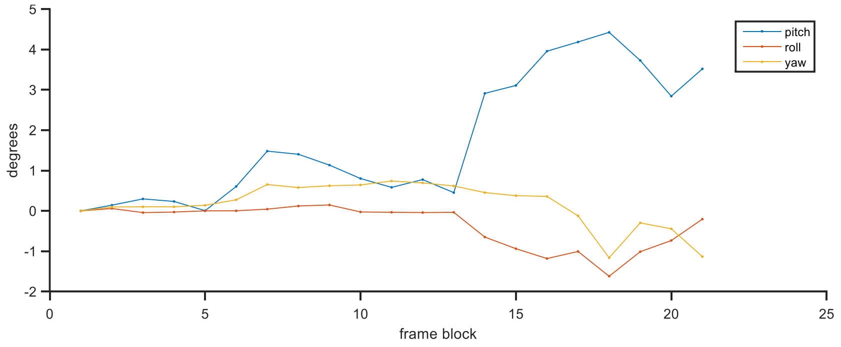

Supplemental Figure 2. Time course for subject movement parameters in subject #5, the first (test) PET measurement. **A.** translation parameters along the x (i.e. left-to-right), y (i.e. posterior-to-anterior), z (i.e. inferior-to-superior) axis. **B.** the corresponding plot of subject rotation parameters around the x (i.e. pitch), y (i.e. roll) and z (i.e. yaw) axis. Heavy subject movement, relative displacement, starts at frame block 14 (corresponding to frame #31 at 48 min post injection).

The realignment parameters were then used to reslice, realign individual time frames of the reconstructed PET image to the position for the reference image at the start of the PET measurement.

3. If subject movement was severe so that the described artefacts (in step 1) were present at visual inspection, then the following procedure was used:

The PET image was re-reconstructed without attenuation correction, resulting in a PET image showing radioactivity counts and not radioactivity concentrations, but with no artefacts. Such images are not useable for the purposes of quantification but amenable for the determination of subject movement. This image reconstructed without attenuation correction

was then evaluated for inter-frame subject movement in the same way as described above (step 2) by using the co-registration algorithm in SPM12.
The obtained movement parameters could now be used to reconstruct the PET image with attenuation correction. For the purpose of reconstruction, the attenuation map was resliced using the inverse of the corresponding frame-wise realignment parameters.
The time frames of the newly reconstructed attenuation-corrected PET image were thus devoid of artefacts but still had the inter-frame subject movement present. Hence the reconstructed time frames were resliced using the obtained realignment parameters to obtain the final movement-corrected, artefact-free PET image. A sample of a PET image corrected using this approach is shown in Supplemental Figure 3.

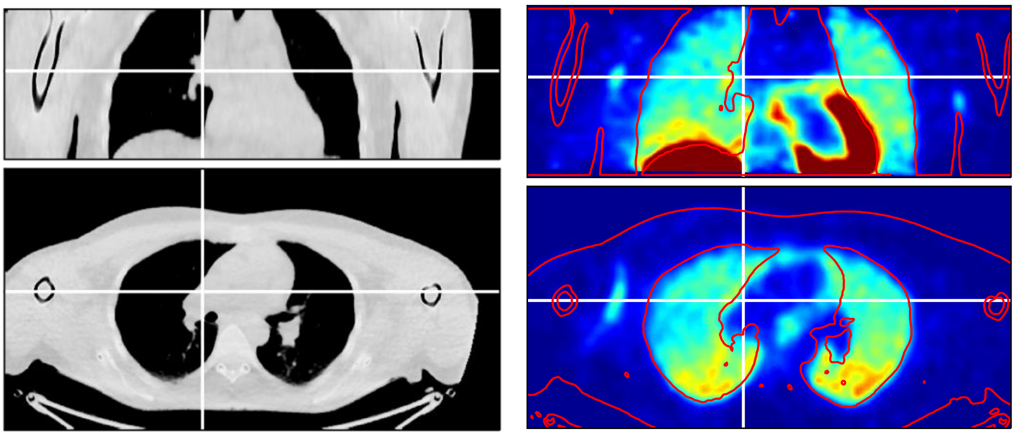

Supplemental Figure 3. Image after correction of inter-frame subject motion. Left: frontal and horizontal slices of CT image for subject #5, second (retest) PET measurement. Right: corresponding slices of the 32nd time frame of the PET image (54 min post injection) after correcting for subject motion. Red contours indicate contrast edges within the CT image showing improved agreement of the lung surface boundaries between CT and PET at this time frame. Furthermore, there are no obvious reconstruction artefacts in the PET frame following the described correction procedure.

Image processing and analysis

Delineation of regions of interest

The delineation of ROIs in PET was achieved using mask images, which contained the indicator value of 1 for image volume elements (voxels) within the corresponding ROI and zero elsewhere. To identify the various structures, the procedure relied on gross anatomical landmarks and the biologically given differences in the temporal sequence by which radioactivity appears at distinct points in the blood circulation (i.e. ventricles of the heart, major vessels) and in organs following I.V. bolus injection. The automated delineation proceeded along the following steps:

1. To obtain an image devoid of the substantial early impact of high radioactivity in blood and to limit the impact of possible subject movements typically appearing more strongly after about 30 min post injection (see supplementary figure 2) a summation (average) PET image corresponding to 12 to 33 min post injection was reconstructed.
2. To reduce image noise this summation image and the corresponding resliced CT image were median filtered (using a 3 × 3 × 3 voxel neighbourhood, i.e. N=27 voxels).
3. The radioactivity concentration of $[^{11}C]$VC002 in heart and liver was higher than in lung tissue. To be able to subsequently control for contamination of the lung signal by spill-in effects from these organs, the summation image from step 2 was used to obtain a preliminary mask image defining the location of heart and liver. In detail, voxels with a radioactivity concentration above an appropriate chosen threshold (see below) were assigned to the mask such that all within-mask

voxels belonged to one or maximum two clusters of contiguous voxels and the total volume of such voxels did not exceed 250 cm^3 since, based on preliminary checks, due to the 16-cm axial FOV of the PET system the combined volume of the topmost tip of the liver and upper part of the left ventricular wall, falling within the FOV of the thoracic PET images, appeared to be below this limit. The threshold yielding a mask satisfying these criteria was obtained iteratively with a starting value corresponding to 90th percentile of the summation image and then gradually increasing it until a match was found.

4. The preliminary combined heart and liver mask was split into separate masks for the heart and liver by assigning the cluster of contiguous voxels on the right side to the liver and the one at the left side to the heart. The heart mask typically corresponded to the left ventricular wall but in some cases also contained the myocardium of the right ventricle.
5. The PET frame corresponding to radioactivity peaking in arteries was then determined as a necessary step for the later steps. Using the heart mask, the TAC for the left ventricular wall was obtained for the initial 100 seconds. The time frame corresponding to the highest radioactivity concentration in this curve was defined as the peak frame. The peak frame was typically the 2nd or 3rd time-frame corresponding to 15-30 seconds post injection.
6. A preliminary ROI mask covering both lungs was obtained by selecting those voxels satisfying a set of operationally defined criteria, i.e. which:
   - had a CT signal corresponding to or below a density of ~760 g/dm^3,
   - had radioactivity levels in the 12-33 min summation image at or above ~0.05 SUV (to avoid locations outside the body or within major airways),
   - had radioactivity concentrations in the period before the peak frame below 25 SUV (to reduce spill-over from large vascular spaces such as vena cava and pulmonary arteries).
   - were at least ~2 voxels away from the combined heart and liver mask (to reduce spill-over effects from those organs).
   - Furthermore, possible remaining tracheal voxels were removed by excluding voxels falling within a 20 × 40 × 50 mm (W × D × H) box placed appropriately in the middle of the preliminary pulmonary mask.
7. Separate whole left and right lung masks were obtained by identifying the two largest clusters of within-mask voxels in the common pulmonary mask.
8. The next part of the delineation procedure aimed to obtain the ROI mask of the aortic arch to generate the image-derived arterial input function. To prepare for that, the mask of the upper mediastinum could now be obtained by selecting voxels between the two lungs and ~15 mm or more above the top of the heart (left ventricular) mask so that all cardiac voxels, even those falling within the left atrium of the heart, would not be included.
9. The upper mediastinal mask was used to calculate an appropriate radioactivity threshold defining the mask of the aortic arch. In detail, the histogram of voxel-wise radioactivity concentrations (i.e. the number of voxels as a function of radioactivity concentration) was calculated in the peak frame (see step 4 above) for voxels within the upper mediastinal mask with raw histogram bins 1 nCi/cm^3 apart. The histogram curve, after smoothing with local regression using weighted linear least squares and a 1st degree polynomial model and a span of 1500 bins, had a peak at very low radioactivity concentrations, corresponding to background, non-vascular voxels, then the curve gradually decreased, finally it levelled out for a shorter or longer range of radioactivity concentration values before it had a slight increase toward the even higher radioactivity values (Supplemental Figure 4). The flat part of the histogram at intermediate high radioactivity levels

occurred due to the fact that within-aorta voxels exhibited gradually increasing radioactivity concentrations in coaxial tubular, bent cylindrical, shells of similar boundary volumes (the decreasing cylinder radius toward higher radioactivity levels was more or less counteracted by the increasing thickness of the cylindrical boundary). The radioactivity value corresponding to the middle of the horizontal section of the flat part of the histogram was taken as the threshold for defining a rough mask of the aortic arch (Supplemental Figure 4). This point corresponds to the lowest point within the histogram curve (i.e. excluding the tails of the histogram).

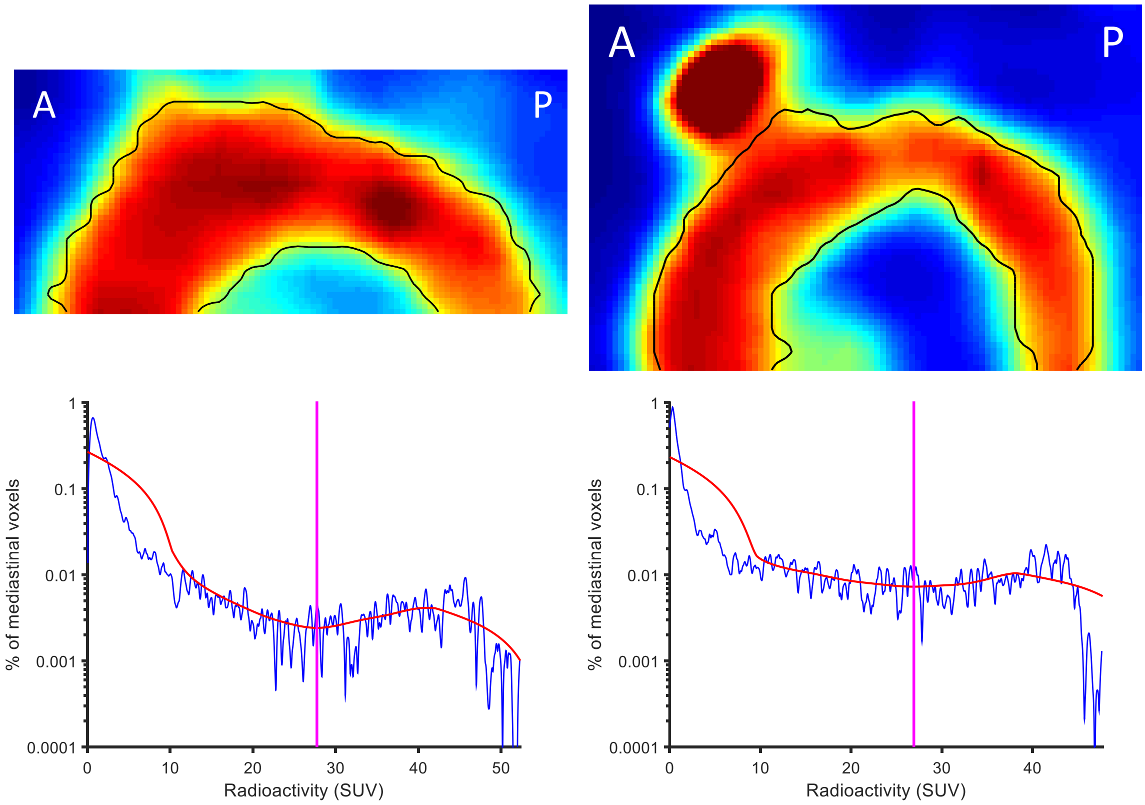

Supplemental Figure 4. Two examples of aortic masks and histograms of voxel-wise radioactivity values within mediastinum. Top left: image of radioactivity concentration of the peak time frame for subject #1, first (test) PET measurement, in a slice through the arch of the aorta along a slightly oblique near-sagittal plane. Image zoomed in to top mediastinum. Black contour line shows the boundary of the aortic mask. Bottom left: histogram curve(s) and threshold for subject #1, first (test) PET measurement. Blue curve shows the raw histogram with light smoothing (with a span of 100 bins) for visualisation, red curve shows the more heavily smoothed curve used for detecting the threshold, and the vertical, magenta line indicates the threshold value corresponding to the lowest point of the flat region of the histogram. Top right: Sample image of the peak time frame for subject #7, first (test) PET experiment indicating contour of aorta mask. The hot spot above the aortic arch is the left brachiocephalic vein through which the bolus of injected radioactivity was delivered from the left arm to the heart. Bottom right: histogram curve(s) and threshold for subject #7, first (test) PET experiment.

10. The rough mask was then refined by keeping only those voxels that had a similar or lower radioactivity in the preceding time frame, then smoothing it (i.e. applying a Gaussian filter with a 2 mm FWHM width and keeping the voxels with a value of at least 0.5 in the mask) and selecting the largest contiguous cluster of within-mask voxels and removing the others. If the total volume of within-mask voxels was above 30 ml, i.e. a practical upper limit for the volume of the arch of the aorta, then the refinement of the raw mask was repeated in such a way that only voxels with negligible radioactivity in the preceding frame (i.e. below 1.5 SUV) were kept before smoothing.
11. Finally, the masks of the whole left and right lungs and the aortic arch from the follow-up occasions were resliced to the space of the baseline data using the co-registration parameters

obtained previously (see above) and only voxels overlapping with the respective baseline masks were retained. The pulmonary and aortic masks were used as ROIs in the subsequent analysis.

Quantitative analyses of [^{11}C]VC-002 binding: compartment models

The PET signal coming from lungs was interpreted according to a compartment model describing the pharmacokinetics of the radioligand in lung tissue. A schematic view of the components and compartments of this model is shown in Supplemental Figure 5 below. Note that in the actual analysis total plasma radioactivity was used so "ligand" in the figure and the following explanation actually refers to both parent radioligand and its radiometabolites with the assumption that they also enter lung tissue.

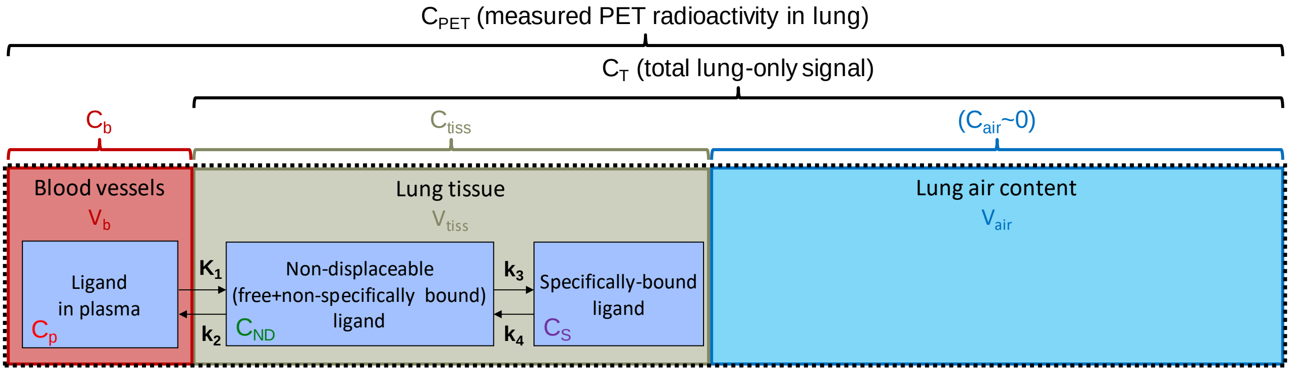

Supplemental Figure 5. PET compartment model for radioligand pharmacokinetics in lungs after i.v. administration

Radioligand entrance into and binding to lung tissue is driven by radioligand present in arterial plasma. Accordingly, the PET pharmacokinetic model operates with a single input compartment (radioligand in arterial plasma) and two compartments in tissue: the non-displaceable (free and non-specifically bound ligand) compartment and the specific compartment. The latter is viewed as corresponding to radioligand bound to the target receptors.

The transition between the compartments is described by first-order pharmacokinetic rate constants K_1 (ml/min/cm^3), k_2 (1/min), k_3 (1/min), k_4 (1/min) [1]. The rate constants determine the change in compartment concentrations as a function of the instantaneous concentration in another compartment at a given time according to the following differential equations:

$$\frac{dC_{ND}(t)}{dt} = K_1 \times C_p(t) - (k_2 + k_3) \times C_{ND}(t) + k_4 \times C_S(t) \qquad \text{Eq. 1}$$

$$\frac{dC_S(t)}{dt} = k_3 \times C_{ND}(t) - k_4 \times C_S(t) \qquad \text{Eq. 2}$$

If the pharmacokinetic system is in true equilibrium, then the left sides of the equations are zero (i.e. there is no change in the concentration in any compartment over time). In this case the equations can be rearranged and combined as the following (dropping the time "(t)" indicator since we refer to equilibrium concentrations):

$$\frac{C_{tiss}}{C_p} = \frac{K_1}{k_2} \times \left(1 + \frac{k_3}{k_4}\right) \coloneqq V_T \qquad \text{Eq. 3}$$

This ratio is referred to as either the partition coefficient or the total volume of distribution (V_T), and it gives a measure of the total binding capacity of the tissue for the radioligand.

Air content correction requires estimation of V_{air}, which is technically possible using the CT image. However, it is an additional source of error and may be inadequate especially when using CT and PET images without respiratory gating information. At the same time, the non-corrected V_T values (i.e. corresponding to $\frac{C_T}{C_p}$ instead of $\frac{C_{tiss}}{C_p}$) should be more stable and be anyhow acceptable for future purposes of estimating drug-induced receptor occupancy if V_{air} can be assumed to be fairly constant across the baseline and pharmacological challenge conditions. Accordingly, in the present work we used the V_T values with no air content correction ($V_T = \frac{C_T}{C_p}$).

V_T has the unit of ml/cm^3: i.e. it gives the volume of blood (in ml) required to match the amount of radioligand molecules present in one unit of or lung-only (or tissue-only) volume (in cm^3). Thus, it has a connection to the traditional pharmacokinetic volume of distribution, while distinct from it: multiplying the V_T value with the total volume of the target organ will give the ml of plasma necessary to account for the total amount of radioligand in that organ. It is good to point out that larger variations of individual rate constants K_1 to k_4 do not necessarily mean a similarly large variation in the composite parameter V_T: the latter can be rather stable even while individual rate constants vary considerably.

If the rate of exchange between the non-displaceable and the specific compartment is rapid then the pharmacokinetic system can be approximated with a single tissue compartment with two rate constants: K_1' and k_2', accounting for the overall rate of exchange between plasma and tissue. Note that these rate constants are not numerically equal to K_1 and k_2 of the two-tissue compartment model when fitted to the same experimental data. V_T in case of the one-tissue compartment model can simply be calculated as:

$$V_T' = \frac{K_1'}{k_2'} \qquad \text{Eq. 4}$$

RESULTS

Plots showing individual lung and arterial plasma time-radioactivity curves in Supplemental Fig. 6. The lung data are shown both for total lung and lung tissue only (i.e. corrected for pulmonary blood radioactivity content). Plasma curves show total radioactivity, i.e. not corrected for radioactive metabolites.

Detailed results of quantification of [^{11}C]VC002 binding to mAChRs in the lungs is presented in Supplemental Table 1.

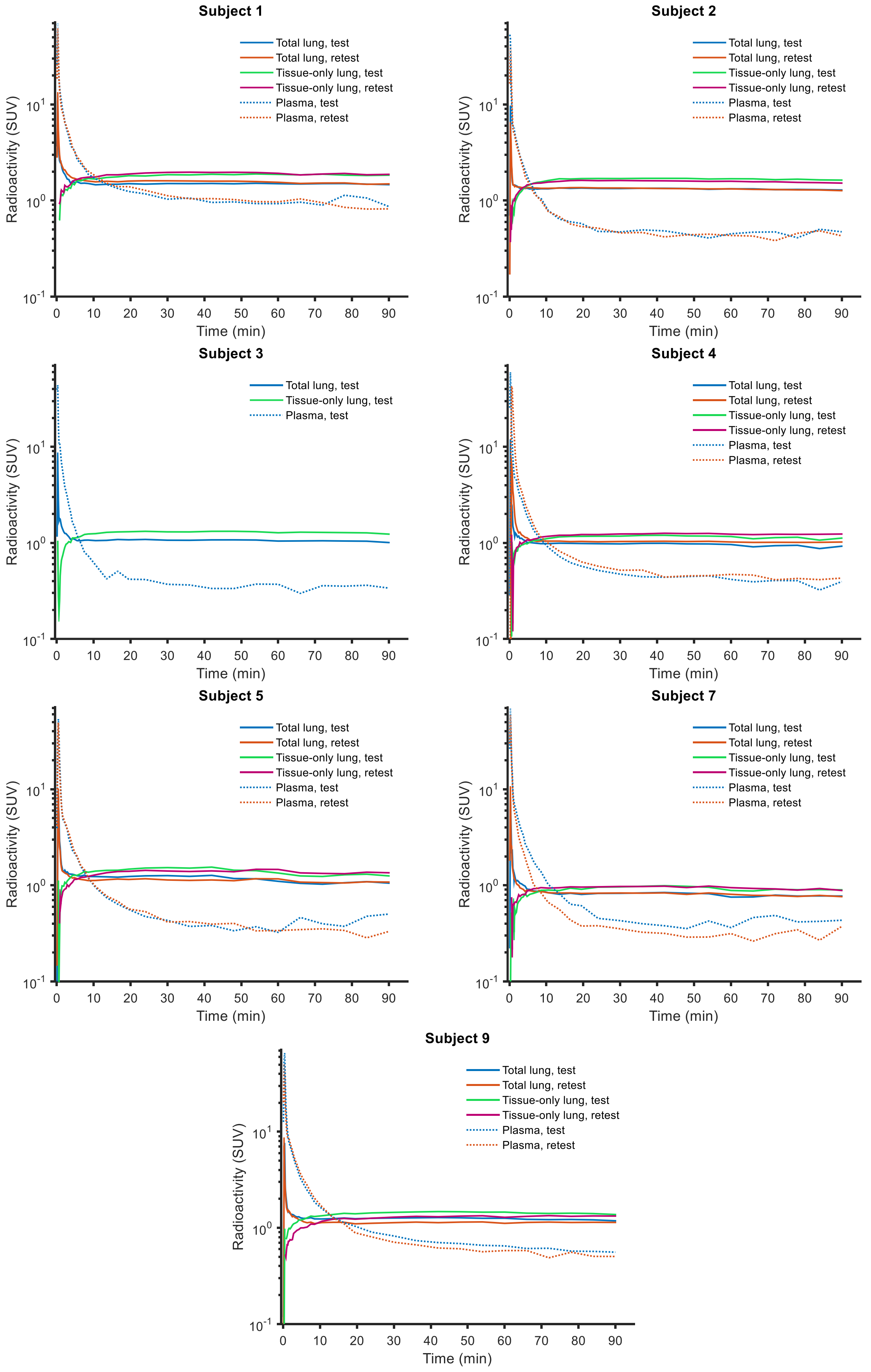

Supplemental Figure 6. Individual time-radioactivity curves in lung and arterial plasma at test and retest

Subject	Test (1), retest (2)	V_b (%)		K_1 (ml/cm^3/min)			k_2 (1/min)		K_1/k_2 (ml/cm$_3$)		k_3 (1/min)	k_4 (1/min)	k_3/k_4	V_T (ml/cm$_3$)***				MO****	AIC*****		2TCM vs. 1TCM F-test******	
		2TCM	DEPICT**	1TCM	2TCM	DEPICT**	1TCM	2TCM	1TCM	2TCM	2TCM	2TCM	2TCM	1TCM	2TCM	MLLogan	DEPICT**	DEPICT	1TCM	2TCM	F-statistic	p-value
1	1	27.4	23.1	0.03	0.03	0.03	0.02	0.14	1.9	0.2	41.37	5.21	7.9	1.9	1.9	1.9	1.8	1	233.2	237.2	-1.9×10^{-5}	1
	2	28.0	23.6	0.03	0.04	0.03	0.02	0.04	1.8	0.8	0.10	0.08	1.2	1.8	1.9	1.8	1.9	1	235.1	237.4	0.77	0.47
2	1	23.5	11.3	0.04	0.04	0.04	0.01	0.10	3.5	0.4	42.81	5.44	7.9	3.5	3.5	3.4	3.5	1	184.9	188.9	2.2×10^{-4}	1
	2	20.0	10.3	0.05	0.05	0.06	0.01	0.03	3.2	1.9	0.06	0.08	0.8	3.2	3.4	3.4	3.4	2	145.0	125.2	14.75	2.3×10^{-5}
3	1	22.4	12.0	0.03	0.03	0.03	0.01	0.11	3.8	0.3	41.76	3.68	11.4	3.8	3.8	3.5	3.1	1	191.3	195.3	-8.3×10^{-6}	1
4	1	22.3	5.3	0.03	0.03	0.04	0.01	0.16	2.5	0.2	24.06	1.79	13.5	2.5	2.5	2.4	2.3	1	257.5	261.5	-4.5×10^{-6}	1
	2	21.9	18.2	0.03	0.03	0.03	0.01	0.02	2.5	1.2	0.07	0.05	1.3	2.5	2.7	2.6	2.7	1	235.5	239.0	0.25	0.78
5	1	21.0	18.3	0.03	0.03	0.03	0.01	0.09	3.0	0.4	48.93	6.87	7.1	3.0	3.0	2.7	2.8	1	207.3	211.3	-2.1×10^{-6}	1
	2	23.4	19.9	0.03	0.03	0.03	0.01	0.08	3.4	0.4	25.55	3.14	8.1	3.4	3.4	3.3	3.4	1	232.2	236.2	-2.3×10^{-5}	1
6	1	19.2	16.5	0.02	0.02	0.02	0.01	0.18	2.2	0.1	30.51	1.40	21.8	2.2	2.2	2.0	2.0	1	146.3	150.3	-4.9×10^{-5}	1
	2	18.4	12.6	0.02	0.02	0.02	0.01	0.20	2.7	0.1	47.08	1.96	24.0	2.7	2.7	2.7	2.5	1	247.1	251.1	4.5×10^{-7}	1
7	1	17.3	15.6	0.02	0.02	0.02	0.01	0.04	1.9	0.7	0.10	0.05	2.1	1.9	2.1	2.0	2.1	1	214.9	212.2	3.26	0.0501
	2	20.2	16.6	0.02	0.02	0.02	0.01	0.01	2.3	1.6	0.02	0.03	0.6	2.3	2.6	2.3	2.4	1	107.3	97.9	7.15	2.5×10^{-3}
Mean ±SD; CV%	1 (N=6)*	21.8 ±3.5; 16%	15.0 ±6.1; 41%	0.03 ±0.01; 29%	0.03 ±0.01; 27%	0.03 ±0.01; 36%	0.01 ±0.00; 23%	0.12 ±0.05; 45%	2.5 ±0.7; 26%	0.3 ±0.2; 65%	31.30 ±17.73; 57%	3.46 ±2.73; 79%	10.0 ±6.8; 67%	2.5 ±0.7; 26%	2.5 ±0.6; 25%	2.4 ±0.6; 24%	2.4 ±0.6; 26%	1 (1 – 1)	207.4 ±38.7; 19%	210.2 ±38.5; 18%		
	2 (N=6)	22.0 ±3.4; 16%	16.9 ±4.9; 29%	0.03 ±0.01; 34%	0.03 ±0.01; 35%	0.03 ±0.01; 42%	0.01 ±0.00; 37%	0.06 ±0.07; 109%	2.6 ±0.6; 23%	1.0 ±0.7; 70%	12.15 ±19.92; 164%	0.89 ±1.34; 151%	6.0 ±9.3; 154%	2.6 ±0.6; 23%	2.8 ±0.6; 20%	2.7 ±0.6; 22%	2.7 ±0.6; 22%	1 (1 – 2)	200.4 ±58.9; 29%	197.8 ±67.6; 34%		
Mean VAR; min–max	(N=6)	8.4%; 1.8%–16.1%	27.1%; 2.1%–109.8%	11.6%; 5.4%–22.3%	14.2%; 0.2%–22.1%	23.0%; 1.1%–52.5%	15.4%; 0.0%–27.5%	81.1%; 7.4%–148.6%	11.2%; 0.1%–22.3%	83.0%; 2.1%–148.6%	140.5%; 42.7%–199.4%	122.8%; 33.4%–194.4%	100.9%; 9.6%–164.3%	11.2%; 0.1%–22.3%	12.0%; 0.2%–23.2%	12.1%; 1.8%–28.1%	12.4%; 1.1%–24.4%	N/A	N/A	N/A	N/A	N/A

1TCM: one-tissue compartment model; 2TCM: two-tissue compartment model; DEPICT: data-driven estimation of parametric images based on compartmental theory; MLLogan: multi-linear variant of Logan's graphical analysis; MO: model order; SD: standard deviation: CV, coefficient of variation; VAR: absolute variability; AIC: Akaike information criterion.

* Summary statistics for test occasion are based only on those 6 subjects who also had retest PET results (i.e. excluding subject #3)

** Parameters for DEPICT indicate voxel-wise averages for voxels within the whole lung ROI (except MO, see below)

*** V_T in case of 1TCM denotes (is the same as) K_1/k_2, and in case of 2TCM it equals $K_1/k_2\times(1+k_3/k_4)$

**** Model order values shown are voxel-wise median values for voxels within the whole lung ROI; summary statistics indicate across subject median and range in parentheses

***** Colour coding indicates the compartment model with lower AIC for easier visual assessment

****** Colour coding of F-statistic and p-values indicates that 2TCM was significantly better than 1TCM (at the α=5% level) according to 1-tailed (right-tailed) F-test; black indicates no significant improvement of 2TCM over 1TCM

Supplemental Table 1. Results of kinetic analysis showing individual and summarized micro- and macro-parameters for [^{11}C]VC-002 binding in the lungs (examination of 90 min), as well as results of statistical comparison between 1TCM and 2TCM

REFERENCES

1. Lammertsma AA, Bench CJ, Hume SP, Osman S, Gunn K, Brooks DJ, et al. Comparison of methods for analysis of clinical [11C]raclopride studies. J Cereb Blood Flow Metab. 1996;16:42–52.
